# The Association Between Indoor Air Pollution and Lung Cancer Risk in a Chinese Population

**DOI:** 10.1155/ina/9937960

**Published:** 2025-02-27

**Authors:** Fang Fang, Jin-Yi Zhou, Claire H. Kim, Zi-Yi Jin, Xing Liu, Liming Li, Lina Mu, Ming Wu, Jin-Kou Zhao, Zuo-Feng Zhang

**Affiliations:** 1 Department of Epidemiology, Fielding School of Public Health, University of California at Los Angeles (UCLA), Los Angeles, California, USA; 2 Department of Non-Communicable Chronic Disease Control, Jiangsu Provincial Center for Disease Control and Prevention, Nanjing, Jiangsu, China; 3 Department of Rheumatology and Immunology, Nanjing University Medical School Affiliated Nanjing Drum Tower Hospital, Nanjing, Jiangsu, China; 4 Department of Epidemiology, School of Public Health, Fudan University, Shanghai, China; 5 Department of Epidemiology and Biostatistics, Peking University School of Public Health, Beijing, China; 6 Department of Epidemiology and Environmental Health, State University of New York at Buffalo, Buffalo, New York, USA

**Keywords:** environmental tobacco smoking, indoor air pollution, lung cancer, sex, tobacco smoking

## Abstract

Though indoor air pollution (IAP) is associated with elevated lung cancer risk, an integrated measure is imperative to thoroughly investigate this association. The interplay between sex and IAP on lung cancer remains unclear. We conducted a population-based case-control study in Jiangsu Province, China, from 2003 to 2010, with 2871 lung cancer cases and 8019 controls. Exposures and covariates information were collected via in-person interviews using a standardized questionnaire. An integrated weighted risk score (WRS), accounting for the effect sizes of each source of IAP, was introduced. Unconditional logistic regression was employed to estimate adjusted odds ratios (aORs) and their 95% confidence intervals (CIs). Interactions between sex and IAP by tobacco smoking status were evaluated. Environmental tobacco smoking (ETS) (aOR = 1.54, 95% CI: 1.40, 1.69), poor ventilation (aOR = 1.18, 95% CI: 1.07, 1.30), and coal used for cooking (aOR = 1.27, 95% CI: 1.15, 1.41) were associated with lung cancer. Dose-response relationships between lung cancer and WRS were observed, with *p* for trend less than 0.001. aOR for individuals at the highest quartile of the WRS of IAP was 1.74 (95% CI: 1.52, 2.00) compared to the lowest quartile. The associations were more profound among never-smokers than ever-smokers. Females tended to be more vulnerable to IAP, and sex interacted with IAP beyond multiplicativity on the odds scale. IAP is associated with lung cancer, with a stronger impact among never-smokers. An interaction between IAP and sex was observed. These results underscore the importance of controlling IAP, especially ETS in order to reduce the risk of lung cancer.

## 1. Introduction

Lung cancer is the most commonly diagnosed cancer and the leading cause of cancer deaths, with approximately 2.4 million new cases and 1.8 million mortalities globally in 2022 [1]. The trend of tobacco use, a major risk factor of lung cancer, has changed disproportionately across different regions and between sexes, resulting in varying lung cancer burdens in different populations [1–6]. In China, over 1 million new lung cancer cases and 733,000 deaths due to lung cancer occurred in 2022, accounting for 42.8% and 40.4% of all occurrences in the world, respectively [1]. Among new cases, about 659,000 cases were males, and about 402,000 cases were females [1]. Moreover, a more than 4.5 times increase in lung cancer incidence has been reported over the past three decades [7], particularly among females [8]. The high incidence and mortality of lung cancer in China can be primarily attributable to the widespread use of tobacco such as active and passive smoking, as there are more than 300 million smokers and over 700 million non-smokers who are exposed to environmental tobacco smoking (ETS) daily [9]. Despite a steady decline in smoking in both sexes, smoking prevalence remains significantly higher among Chinese males (50.7%) compared to females (1.9%) in 2018 [10]. Though tobacco smoking is the most important risk factor for lung cancer, the difference in tobacco smoking prevalence between males and females does not fully explain the sex disparity in lung cancer. Therefore, it is crucial to investigate other risk factors, such as air pollution, especially among females in Asian populations, whose tobacco smoking prevalence is very low [1, 11, 12]. Furthermore, indoor air pollution (IAP) warrants special attention, given the substantial time Chinese females spend in their homes due to household responsibilities. The World Bank Group data showed that Chinese females spent 15.3% of their time on unpaid domestic and care work, while this work took Chinese males 5.9% of their time [13].

IAP is a risk factor for lung cancer, especially among those who never smoke [1, 14, 15], containing carcinogens, such as benzene, formaldehyde, particulate matter, asbestos, and certain volatile organic compounds [16]. Major sources of IAP include combustion of cooking and heating fuel, ETS, penetration of ambient air pollution, building materials, and furnishings [17–19]. Although diluted in air, ETS, or secondhand tobacco smoke, contains the same toxic substances and carcinogens that are inhaled by the smoker [20]. The International Agency for Research on Cancer (IARC) estimated the excess risk of lung cancer due to exposure to secondhand smoke from a spouse who smokes is approximately 30% for men and 20% for women [21]. Positive associations between ETS exposure and lung cancer risk have been reported, though heterogeneity exists based on smoking status, sex, and location of exposure [22–26]. Other than ETS exposure, solid fuel used for cooking and heating and cooking fumes contribute largely to the elevated IAP or household air pollution, producing pollutants such as particulate matter, nitrogen oxides, and volatile organic compounds [17]. In 2020, approximately 3.2 million deaths were attributed to IAP in the world [19], and close to 1 million deaths were due to IAP in China [27]. IAP induced by biomass fuels or solid fuels use was reported to be associated with an increased risk of lung cancer, but the associations varied by type of fuel, sex, and subtype of lung cancer [23, 28–32]. In addition, household ventilation has been shown to protect individuals from IAP in previous studies [31, 33–35].

Although previous studies have concluded a positive association between IAP and lung cancer, the methods for assessing IAP exposure have varied. Most studies have examined associations between lung cancer and individual factors of IAP [22–26, 28–35], while other studies have assessed IAP as a binary variable [30], the sum of total IAP factors [35], or concentrations of pollutants either in the household [36] or as biomarkers [37]. These varying methods are not always adaptable to every study. For example, questionnaire-based studies cannot measure the concentration of indoor air pollutants and must rely on participant responses. Most studies have focused on individual IAP factors or treated IAP exposure as a binary variable, overlooking the varying impacts of different IAP factors on lung cancer risk. Assessing multiple IAP factors may offer a more comprehensive depiction of the actual exposure level individuals face, but a simple index encompassing a total number of exposure sources neglects the fact that each individual IAP factor has a different impact on lung cancer risks.

To address the limitations of these existing methods, we introduce an integrated weighted risk score (WRS), which is computed based on the participant’s binary response to individual IAP factors. Instead of treating each IAP factor alike or simply introducing an overall binary IAP measure, we suggest accounting for the effect size of each exposed IAP factor to assess one’s risk due to exposure to multiple IAP factors. This could potentially improve the accuracy of IAP exposure assessment when only questionnaire data is available and could better assess the association between exposure to multiple IAP factors and various health outcomes.

While previous studies have demonstrated a positive association between lung cancer and IAP, it remains important to investigate this relationship using a refined measure of IAP that not only assesses multiple factors simultaneously but also accounts for the effect size of each factor. Furthermore, given the detected heterogeneity across smoking status and sex, this population-based case-control study examines these associations within a rural Chinese population, employing WRS, a novel metric, to identify high-risk populations and provide insights into the role of sex in this relationship.

## 2. Material and Methods

**Figure 1:**
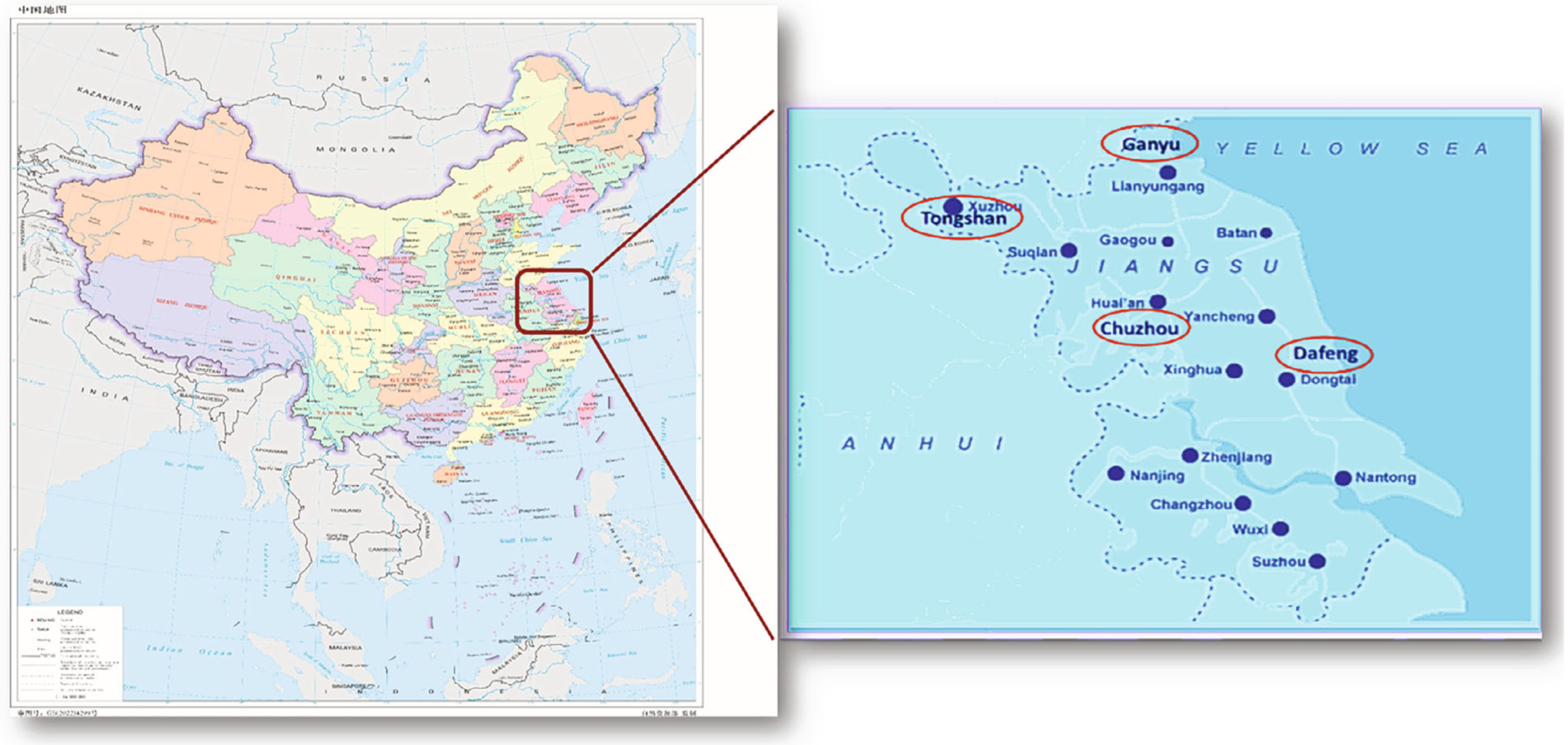
Map of Jiangsu Four Cancer Study.

The Jiangsu Four Cancers (JFC) study is a population-based case-control study of incident cancers of the lung, liver, stomach, and esophagus, conducted in four counties (Chuzhou, Dafeng, Ganyu, and Tongshan) of Jiangsu Province, China (Figure 1), between 2003 and 2010 [38]. This study was approved by the Human Subject Protection Committees of the Jiangsu Provincial Center for Disease Control (CDC) and the Institutional Review Boards of the University of California at Los Angeles (UCLA). Informed consent was obtained from each participant. The study details have been published previously [38]. Briefly, to be eligible for this study, participants needed to have resided in their respective counties for at least 5 years. Incident lung cancer cases aged 18 years or older were identified through the local population-based cancer registries. Most cases (64.5%) were diagnosed by computer tomography. A small portion (14.8%) were diagnosed with pathological examination due to the study sites being in underdeveloped rural areas. Controls were individuals with no history of cancer, recruited randomly from the same county as the cases, using the demographic database of each county. Controls were matched to cases by age (± 5 years) and sex. However, we broke the individual matching and included controls originally matched to other cancer types in the analysis. Participation rates were 42.6% among cases and 86.9% among controls. The total study sample included 2871 cases and 8019 controls. For 1097 of the cases, a family member served as a proxy respondent. Upon enrollment, trained interviewers administered a standardized questionnaire in person, which gathered detailed information about sociodemographic characteristics, medical history, family history of cancer, occupational history, tobacco smoking, dietary practices, and exposures to other potential risk or protective factors for cancer [38]. The questionnaire included specific questions about exposure to ETS, assessing the location of exposure (never, home only, work only, or both home and work), duration of exposure (in years), and intensity of exposure (never, light as 1–2 days per week, medium as 3–5 days per week, or heavy as more than 5 days per week). ETS was further characterized as a binary variable (never or ever). Participants also provided responses about other individual sources of IAP, including high-temperature cooking oil (no or yes), coal used for cooking (no or yes), and solid fuel used for heating (no or yes). Additionally, information on household ventilation (good or poor) was collected.

**Figure 2:**
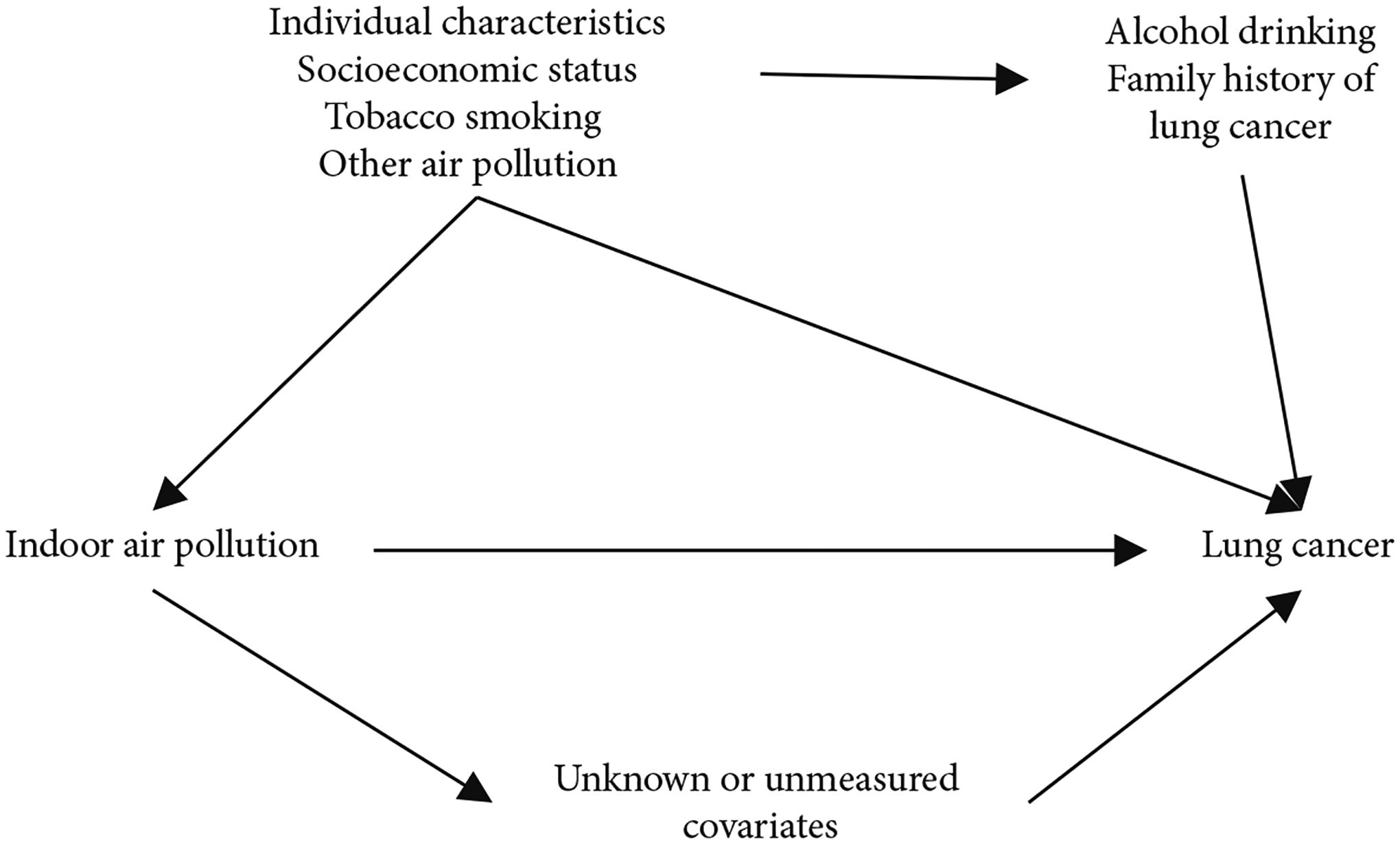
Directed acyclic graph. Indoor air pollution (IAP) includes exposure to environmental tobacco smoking, hot cooking oil, coal used for cooking, solid fuel used for heating, and poor ventilation. Individual characteristics include age, sex, and education. Socioeconomic status includes household income 10 years ago and county of residence. Other air pollution includes other IAP sources or factors affecting IAP.

The distributions of demographic factors between cases and controls were compared using the chi-squared test. Missing values were imputed using multiple imputations [39]. Multivariate unconditional logistic regression was used to estimate adjusted odds ratios (aORs) and their 95% confidence intervals (CIs) for the associations between each individual source of IAP and lung cancer. Analyses were performed among all participants and were stratified by tobacco smoking status (ever or never). Potential confounders were selected based on their association with lung cancer risk and IAP and the directed acyclic graph is shown in Figure 2. The Pearson correlation between independent variables was examined. The models were adjusted for age, sex, income 10 years ago, education, county of residence, alcohol drinking, family history of lung cancer, tobacco smoking pack-years (for all participants and ever-smokers), and tobacco smoking status (for all participants only). Additionally, the models were mutually adjusted for other factors of IAP if applicable.

To account for the presence of multiple IAP factors in the household, two summary measures were computed. The first measure is the total number of IAP factors. This was calculated by summing up each individual factor of IAP exposure, as shown in the following equation: Total number of IAP = ∑IAP_*i*_, where IAP_*i*_ represents the exposure to individual IAP factors, including poor ventilation (1 = *yes* or 0 = *no*), hot cooking oil (1 = *yes* or 0 = *no*), coal used for cooking (1 = *yes* or 0 = *no*), solid fuel used for heating (1 = *yes* or 0 = *no*), and ETS (1 = *ever* or 0 = *never*). In addition to the total number of IAP factors, we introduced a novel measure, the WRS of IAP, which accounts for the effect size of each individual IAP factor. The WRS is calculated using the equation: WRS = ∑*β*_*i*_ × IAP_*i*_, where *β*_*i*_ is the coefficient from the logistic regression model among all participants that included each individual IAP factor and was fully adjusted for all covariates. To examine the associations between lung cancer and the total number of IAP and the WRS, unconditional logistic regression was used. These models were adjusted for the same set of potential confounders, including age, sex, income 10 years ago, education, county of residence, alcohol drinking, family history of lung cancer, tobacco smoking pack-years (for all participants, and ever-smokers), and tobacco smoking status (for all participants only).

To investigate the effect modification of sex, the associations between IAP measures and lung cancer risk among males and females were examined separately. This was done using multivariate unconditional logistic regression adjusting for age, income 10 years ago, education, county of residence, alcohol drinking, family history of lung cancer, tobacco smoking pack-years (for all participants and ever-smokers), tobacco smoking status (for all participants only), and mutually adjusted for other IAP factors if applicable. In addition, interactions between IAP exposures and sex were examined by including interaction terms between sex and IAP measures in the multivariate unconditional logistic regression. The IAP exposures considered included ETS, dichotomized total number of IAP factors (≤ 3 or ≥4), and dichotomized WRS based on the median among all controls. To assess the interaction between sex and IAP measures on lung cancer risk, the relative excess risk due to interaction (RERI) was computed for the additive scale, and the ratio of odds ratio (ROR) was computed for the multiplicative scale [40, 41]. All statistical analyses were performed using SAS 9.4 (Cary, North Carolina).

## 3. Results

A total of 2871 cases, including 1793 ever-smokers and 1078 never-smokers, and 8019 controls, including 3727 ever-smokers and 4292 never-smokers, were eligible and included in this study. The distributions of demographic characteristics by smoking status are presented in Table 1. Among ever-smokers, the distributions of sociodemographic characteristics were similar between cases and controls. However, among never-smokers, cases were more likely to be female, less educated, and never alcohol drinkers compared to controls. Additionally, a higher proportion of missing values in income was observed among cases (Table 1).

Table 2 presents the associations between IAP and lung cancer among all participants, stratified by smoking status. When examining individual IAP factors, ETS exposure was associated with lung cancer among all participants (aOR = 1.54, 95% CI: 1.40, 1.69) after adjusting for potential confounders. Importantly, this association between ETS and lung cancer risk was stronger among never-smokers (aOR = 1.84, 95% CI: 1.58, 2.15) compared to ever-smokers (aOR = 1.32, 95% CI: 1.17, 1.49). Moreover, longer duration and stronger intensity of ETS exposure tended to be associated with a further increase in lung cancer risk (*p* for trend < 0.001). In terms of locations of ETS exposure, residential exposure, including both home and work exposure (aOR = 1.73, 95% CI: 1.47, 2.04) and home-only exposure (aOR = 1.57, 95% CI: 1.41, 1.74), was associated with elevated lung cancer risk. However, a marginal association (aOR = 1.18, 95% CI: 0.97, 1.44) was observed between work-only ETS exposure and lung cancer (Table S1). Other than ETS exposure, poor ventilation (aOR = 1.18, 95% CI: 1.07, 1.30) and coal used for cooking (aOR = 1.27, 95% CI: 1.15, 1.41) were associated with an elevated risk of lung cancer among all participants. However, the associations between lung cancer and high-temperature cooking oil (aOR = 1.02, 95% CI: 0.93, 1.13) or solid fuel used for heating (aOR = 1.09, 95% CI: 0.97, 1.21) were not observed. Similar associations were observed after stratification by smoking status, except that solid fuel used for heating was associated with increased lung cancer odds (aOR = 1.24, 95% CI: 1.08, 1.44) among ever-smokers.

When considering multiple IAP factors, a simple index of total number of IAP exposures suggested that exposure to four or more IAP factors was associated with an increased risk of lung cancer, and a dose-response relationship was observed regardless of smoking status. To further investigate the association between lung cancer and multiple IAP factors, we introduced a novel measure called the WRS, which integrates the effect size of each individual source of IAP. Compared to those with WRS below the median among all controls (WRS = 0.4049), individuals with a higher WRS experienced increased odds of lung cancer with an aOR of 1.50 (95% CI: 1.37, 1.65). When categorizing WRS into quartiles, those in the highest quartile (WRS ≥ 0.5823) had the greatest increase in lung cancer risk (aOR = 1.74, 95% CI: 1.52, 2.00), compared to those in the third quartile (0.4049 ≤ WRS < 0.5823; aOR = 1.25, 95% CI: 1.08, 1.45) and those in the second quartile (0.1874 ≤ WRS < 0.4049; aOR = 1.02, 95% CI: 0.87, 1.18). A dose-response relationship was demonstrated (*p* for trend < 0.001). Assuming linearity, each interquartile range (IQR) increase (IQR = 0.3949) in WRS was associated with a 48% increase in lung cancer odds (aOR = 1.48, 95% CI: 1.38, 1.60). These associations were consistently observed after stratification by smoking status, with a slightly greater increase in lung cancer odds associated with IAP observed among never-smokers (Table 2).

**Figure 3:**
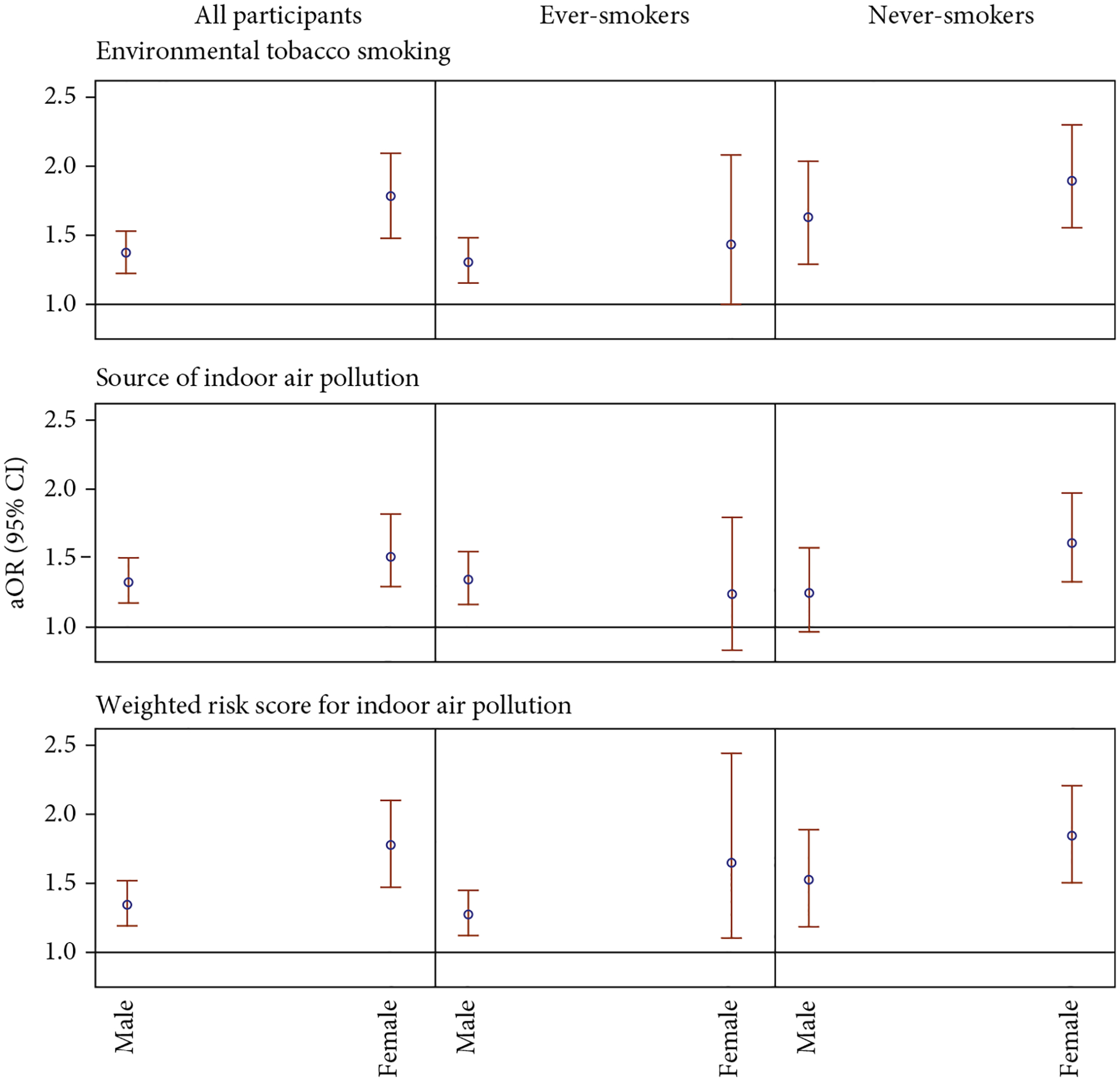
Associations between indoor air pollution (IAP) and lung cancer by sex. Indoor air pollution IAP = 0 defined as not exposed to environmental tobacco smoking (ETS), exposed to three or less sources of IAP (including poor ventilation, hot cooking oil, coal used for cooking, solid fuel used for heating, and ETS exposure), or with a weighted risk score for IAP (sum of effect estimates of poor ventilation, hot cooking oil, coal used for cooking, solid fuel used for heating, and ETS exposure in the model among all participants; the median among all controls is 0.4049) below median. IAP = 1 defined as exposed to ETS, exposed to four or more sources of IAP, or with a weighted risk score for IAP at median or above.

**Figure 4:**
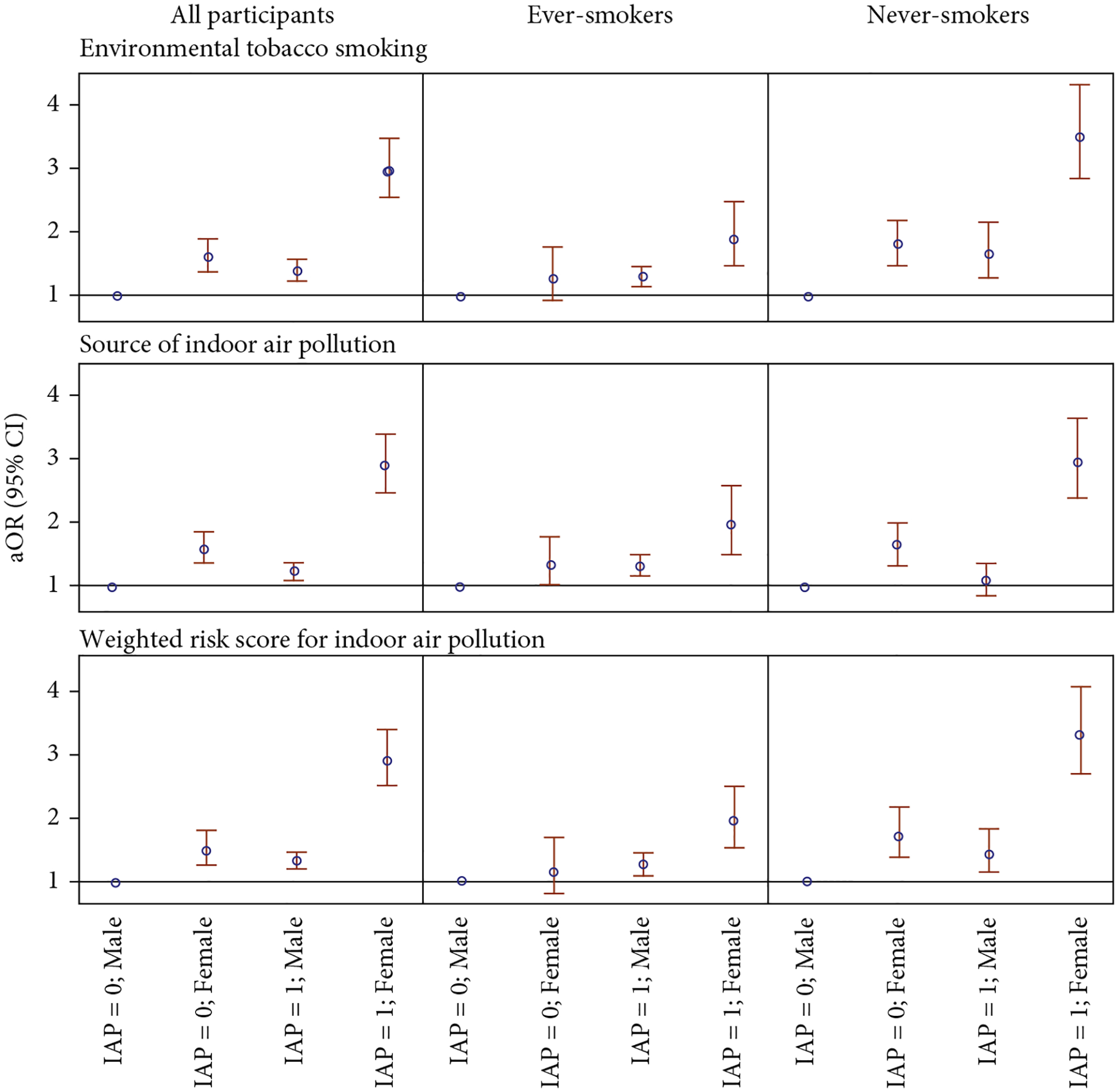
Joint effects of indoor air pollution (IAP) and sex on lung cancer. Indoor air pollution (IAP) = 0 defined as not exposed to environmental tobacco smoking (ETS), exposed to three or less source of IAP (including poor ventilation, hot cooking oil, coal used for cooking, solid fuel used for heating, and ETS exposure), or with a weighted risk score for IAP (sum of effect estimates of poor ventilation, hot cooking oil, coal used for cooking, solid fuel used for heating, and ETS exposure in the model among all participants; the median among all controls is 0.4049) below median. IAP = 1 defined as exposed to ETS, exposed to four or more sources of IAP, or with a weighted risk score for IAP at median or above.

We also investigated whether sex modifies the associations between ETS or IAP and lung cancer (Figure 3 and Table S2) and whether it interacts with ETS or IAP (Figure 4 and Table S3), potentially amplifying the risk of lung cancer. As shown in Figure 3 and Table S2, results suggested that sex is an effect modifier for the association between IAP, including ETS, and lung cancer, with females displaying a greater elevation in lung cancer odds. Specifically, the aOR associated with having a WRS at or above the medians for females was 1.77 (95% CI: 1.49, 2.11), while for males, it was 1.35 (95% CI:1.20, 1.52). This sex disparity in lung cancer odds associated with IAP was observed among both ever-smokers and never-smokers, with larger aORs among never-smokers in both sexes, consistent with the main effects reported in Table 2. However, much wider CIs were observed after further stratification. The associations between lung cancer and ETS and the total number of IAP exposures were similar to those between lung cancer and WRS.

To further investigate the role of sex in the association between IAP and lung cancer, Figure 4 and Table S3 illustrate the interaction between sex and IAP. Among all participants, females with a WRS of IAP at the median or above experienced the greatest increase in lung cancer odds with aOR_11_ of 2.90 (95% CI: 2.50, 3.36), compared to males with a similar WRS (aOR_10_ = 1.34, 95% CI: 1.20, 1.50) and females with a lower WRS (aOR_01_ = 1.50, 95% CI: 1.27, 1.79). The interactions on the additive scale and the multiplicative scale were assessed by RERI (1.05, 95% CI: 0.66, 1.44) and ROR (1.44, 95% CI: 1.17, 1.76), respectively. Therefore, our results indicate an interaction beyond multiplicative on the odds scale between sex and WRS concerning the risk of lung cancer. This interaction remains after stratification by smoking status and is more pronounced among never-smokers. Moreover, sex also interacts with ETS and the total number of IAP exposure beyond the multiplicative scale. These findings suggest that IAP exposure poses additional risks of lung cancer in females.

## 4. Discussion

In this population-based case-control study, IAP was associated with elevated lung cancer risk among both ever-smokers and never-smokers. Aside from confirming previous findings that lung cancer risk can be attributed to individual IAP factors such as ETS exposure, poor ventilation, hot cooking oil, and coal used for cooking, the combined effects of multiple IAP factors, particularly when assessed using the WRS based on effect sizes, were found to increase lung cancer risk in a dose-response manner. These associations were more profound among never-smokers compared to ever-smokers. Furthermore, sex modified the associations between IAP and lung cancer, with females experiencing more pronounced increases in lung cancer risk when exposed to IAP. Sex interacted with IAP on lung cancer beyond multiplicativity on the odds scale.

ETS contains carcinogens, including benzene, 1,3-butadiene, benzo[a]pyrene, and 4-(methylnitrosamino)-1-(3-pyridyl)-1-butanone [21], and leads to carcinogenesis through pathways such as genotoxicity [42–44] and increasing DNA adducts of tobacco-related carcinogens [45, 46]. Consistent with findings from previous epidemiology studies [21–26], a positive association between ETS and lung cancer was observed among both never-smokers and ever-smokers in this study. The aOR estimated by the current study (aOR = 1.54, 95% CI: 1.40, 1.69) was similar to the odds ratio (OR) estimated by a meta-analysis using Chinese studies (OR = 1.64, 95% CI: 1.34, 2.01) [26]. Though the effects estimated by the current study were consistently stronger than those estimated by the International Lung Cancer Consortium study (ILCCO) (aOR = 1.34, 95% CI: 1.24, 1.45), both studies observed a stronger impact of residential exposure and a dose-response relationship between ETS duration and lung cancer risk [25].

Household use of solid fuels and high-temperature cooking, the other major sources of IAP, also produce carcinogens, such as polycyclic aromatic hydrocarbons and particles, both of which are associated with lung cancer [36, 47]. Our results suggested that coal used for cooking elevated lung cancer risks by about 27% regardless of smoking status (Table 2), consistent with estimates from a meta-analysis [23] and a previous study using a subgroup (two of the four counties) of the current study [35]. Additionally, the previous study showed that good ventilation might protect individuals against lung cancer [35], and our results similarly suggested that poor ventilation was associated with increased risks of lung cancer, consistent with a cohort study of never-smoking women conducted in Shanghai, China [31]. Without efficient ventilation, hazardous pollutants cannot be expelled from the household in a timely manner, increasing the likelihood of exposure for residents. However, lung cancer was no longer associated with solid fuel used for heating or high-temperature cooking oil as previously suggested [35], probably due to the heterogeneity among counties.

In addition to examining the associations between lung cancer risk and individual sources of IAP, it is imperative to assess the impact of IAP by considering various factors affecting IAP simultaneously. This approach provides a more comprehensive understanding of an individual’s exposure to IAP in their household. Though estimating the concentrations of different pollutants either in the household [36] or as biomarkers [37] might seem compelling, it can result in a restricted sample size [37] due to the expenses involved and may be vulnerable to the validity of the predictive model and the representativeness of the sampling [36]. Moreover, for many existing studies, such as the current one, where data collection has already been completed, it is not feasible to collect air samples retrospectively. Therefore, investigators have often utilized information from questionnaires by assessing individual sources of IAP emissions and factors influencing IAP [22–26, 28–35]. Using the number of IAP factors represents a convenient approach, revealing a dose-response relationship between the number of pollutant sources and the risk of lung cancer, as observed in our study and a previous investigation [35]. Although counting the number of IAP factors is straightforward and easy to interpret, its accuracy is limited since the risk associated with each IAP factor may differ. To address this limitation, we introduced the measure of the WRS, which considers the effect size of each individual IAP factor. This approach overcomes the shortcomings of a simple count of IAP exposures while retaining the advantages of considering multiple factors simultaneously. Furthermore, the comparability of the number of IAP factors across different studies may be challenging due to variations in assessed exposure. However, with a properly specified WRS, cross-study comparisons become feasible, even when different IAP factors are evaluated. To validate the utility of WRS as an IAP measure, we compared its results with those obtained using the number of factors contributing to IAP and ETS, a well-established IAP source. Consistent findings across all three measures, both in main analyses and stratified analyses, suggest that WRS is a reliable measure of IAP in the present study.

Another important finding of the current study is the sex disparity in the association between IAP and lung cancer risk. Females tended to be more vulnerable to IAP due to their household responsibilities and the intensity and duration of exposure to ETS produced by their smoking household members [13, 19, 21]. After stratifying by sex, females experienced a greater increase in lung cancer risk after exposed to ETS, and this association was more profound among never-smokers, with an aOR of 1.88 (95% CI: 1.55, 2.29, Table S2). Our observation is consistent with the estimated effect in an earlier study among female nonsmokers in China [34]. Additionally, our study reports a more than multiplicative interaction between sex and IAP on lung cancer risk, suggesting that being female and exposed to IAP poses a greater threat of lung cancer. These findings underscore the disparity in lung cancer risk associated with exposure to IAP between sexes. Since sex is not modifiable, these results highlight the importance of IAP control measures, particularly strategies to mitigate exposure to ETS. Consistent with the main effects of IAP shown in Table 2, Figure 3, and Table S2, the interactions were more profound among never-smokers, whose results are less likely to be influenced by active tobacco smoking. Although smoking history has been assessed in detail, measurement error may persist due to unmeasured factors such as the depth of inhalation, potentially leading to residual confounding when evaluating the associations between IAP and lung cancer risk among ever-smokers. Moreover, since tobacco smoking is a dominant risk factor of lung cancer, it may supersede the effect of other risk factors, including the main effect of IAP and its interaction with sex. As a result, the associations between IAP and lung cancer are more readily observed among never-smokers.

This study has several additional limitations. Although cases were identified by a tumor registry and controls were selected randomly from the same county as their matched cases, the participation rate was moderate among cases (42.6%) compared to controls (86.9%). However, selection bias would pose a threat to the validity of our results only if participation depended on the level of IAP. To address the validity of questionnaires completed by proxies, we additionally control proxy interview (Table S4) and further exclude proxy interviewers (Table S5), resulting in similar conclusions to the main analyses. Recall bias might be possible due to the retrospective collection of information through questionnaires, as cases tend to report exposure to IAP more frequently, potentially causing an overestimation of our estimated effects. Measurement error could also arise from self-reported responses to the questionnaire, rather than from objective measurements. Additionally, although we tried to incorporate different IAP factors, we acknowledge that the current study’s WRS may be limited by the factors included in the questionnaire and might still underestimate the threat of IAP due to other unmeasured factors. For instance, the penetration of ambient air pollution is an important source of IAP [18]. However, our study was conducted between 2003 and 2010, a period during which air pollution levels in China were relatively steady [48], indicating limited temporal variation in ambient air pollution during this period. Regarding spatial variation, all participants resided in four counties, and this was controlled for in our model. The spatial variation within each county was not examined but is likely negligible. Furthermore, while the correlation between individual IAP factors in our study is low (Table S6), future studies utilizing the WRS should consider exploring and accounting for the potential interplay among various factors affecting IAP, as needed. Future studies with a more comprehensive assessment of IAP might apply the WRS measure to better understand the effect of IAP on lung cancer or other disease risks. Although participants in this study were more representative compared to studies conducted among never-smokers or among females, the generalizability of the results might still be limited since only four counties were selected and they were all located in the same province. The study population’s demographics, in terms of education and income level, might be very different from the population selected from an urban area. Nevertheless, this is the first study to assess and combine different factors affecting IAP according to their effect sizes. Our results underscore the critical importance of implementing IAP control measures. While promoting the use of clean energy appliances and ensuring adequate ventilation may be particularly relevant in less developed regions, such as rural China, reducing tobacco smoking remains essential in both developed and developing areas. This approach not only lowers lung cancer risk among ever-smokers but also reduces exposure to ETS, benefiting both ever-smokers and never-smokers. Therefore, policies aimed at minimizing ETS exposure and supporting smoking cessation should be prioritized by policymakers.

## 5. Conclusion

This population-based study uses comprehensive data to assess lung cancer risk from IAP, with the novel WRS enhancing precision. Both WRS and traditional IAP measures are linked to increased lung cancer risk, with females showing greater vulnerability. These findings emphasize the need for IAP mitigation, particularly through tobacco control to reduce ETS exposure, benefiting both never-smokers and ever-smokers.

## Supplementary Material

Supplementary material

## Figures and Tables

**Table 1:** Distribution of sociodemographic characteristics of lung cancer cases and controls by tobacco smoking status.

	All participants			Ever smokers			Never smokers		
	Cases, *N* (%)	Controls, *N* (%)	*p* value^a^	Cases, *N* (%)	Controls, *N* (%)	*p* value^a^	Cases, *N* (%)	Controls, *N* (%)	*p* value^a^
Total	2871 (100)	8019 (100)		1793 (100)	3727 (100)		1078 (100)	4292 (100)	
County of residence			< 0.001			< 0.001			< 0.001
Chuzhou	514 (17.9)	1180 (14.7)		319 (17.8)	476 (12.8)		195 (18.1)	704 (16.4)	
Dafeng	624 (21.7)	2536 (31.6)		495 (27.6)	1438 (38.6)		129 (12.0)	1098 (25.6)	
Ganyu	799 (27.8)	2010 (25.1)		534 (29.8)	1246 (33.4)		265 (24.6)	764 (17.8)	
Tongshan	934 (32.5)	2293 (28.6)		445 (24.8)	567 (15.2)		489 (45.4)	1,726 (40.2)	
Sex			0.004			0.305			< 0.001
Male	1,984 (69.1)	5767 (71.9)		1590 (88.7)	3,339 (89.6)		394 (36.5)	2428 (56.6)	
Female	887 (30.9)	2252 (28.1)		203 (11.3)	388 (10.4)		684 (63.5)	1864 (43.4)	
Age (years)			0.504			0.954			0.622
Less than 50	304 (10.6)	884 (11.0)		142 (7.9)	300 (8.0)		162 (15.0)	584 (13.6)	
50–59	635 (22.1)	1794 (22.4)		389 (21.7)	812 (21.8)		246 (22.8)	982 (22.9)	
60–69	931 (32.4)	2565 (32.0)		626 (34.9)	1283 (34.4)		305 (28.3)	1282 (29.9)	
70–79	816 (28.4)	2195 (27.4)		530 (29.6)	1092 (29.3)		286 (26.5)	1103 (25.7)	
80 or above	185 (6.4)	581 (7.3)		106 (5.9)	240 (6.4)		79 (7.3)	341 (7.9)	
Education			< 0.001			0.246			< 0.001
Illiterate	1384 (48.2)	3831 (47.8)		775 (43.2)	1,697 (45.5)		609 (56.5)	2134 (49.7)	
Primary school	976 (34.0)	2515 (31.4)		677 (37.8)	1288 (34.6)		299 (27.7)	1227 (28.6)	
Middle school	402 (14.0)	1320 (16.5)		278 (15.5)	603 (16.2)		124 (11.5)	717 (16.7)	
High school or above	95 (3.3)	335 (4.2)		60 (3.4)	132 (3.5)		35 (3.3)	203 (4.7)	
Missing	14 (0.5)	18 (0.2)		3 (0.2)	7 (0.2)		11 (1.0)	11 (0.3)	
Income 10 years ago (yuans/year)			< 0.001			0.002			0.005
Less than 1000	606 (21.1)	1710 (21.3)		376 (21.0)	832 (22.3)		230 (21.3)	878 (20.5)	
1000–1999	912 (31.8)	2610 (32.6)		562 (31.3)	1250 (33.5)		350 (32.5)	1360 (31.7)	
2000–2999	612 (21.3)	1762 (22.0)		389 (21.7)	802 (21.5)		223 (20.7)	960 (22.4)	
3000 or above	652 (22.7)	1804 (22.5)		417 (23.3)	792 (21.3)		235 (21.8)	1012 (23.6)	
Missing	89 (3.1)	133 (1.7)		49 (2.7)	51 (1.4)		40 (3.7)	82 (1.8)	
Alcohol drinking			0.013			0.542			< 0.001
Never	1463 (51.0)	4303 (53.7)		562 (31.3)	1138 (30.5)		901 (83.6)	3165 (73.7)	
Ever	1408 (49.0)	3716 (46.3)		1231 (68.7)	2589 (69.5)		177 (16.4)	1127 (26.3)	
ETS exposure			< 0.001			< 0.001			< 0.001
Never	1323 (46.1)	4816 (60.1)		757 (42.2)	1855 (49.8)		556 (52.5)	2961 (69.0)	
Ever	1455 (50.7)	3008 (37.5)		1019 (56.8)	1824 (48.9)		436 (40.5)	1184 (27.6)	
Missing	93 (3.2)	195 (2.4)		17 (1.0)	48 (1.3)		76 (7.1)	147 (3.4)	
Location of ETS exposure			< 0.001			< 0.001			< 0.001
Never	1323 (46.1)	4816 (60.1)		757 (42.2)	1855 (49.8)		556 (52.5)	2961 (69.0)	
Home only	943 (32.9)	1947 (24.3)		595 (33.2)	1098 (29.5)		348 (32.3)	849 (19.8)	
Work only	166 (5.8)	454 (5.7)		149 (8.3)	298 (8.0)		17 (1.6)	156 (3.6)	
Both home and work	296 (10.3)	514 (6.4)		237 (13.2)	376 (10.1)		59 (5.5)	138 (3.2)	
Missing	143 (5.0)	288 (3.6)		55 (3.1)	100 (2.7)		88 (8.2)	188 (4.4)	
Duration of ETS exposure (years)			< 0.001			< 0.001			< 0.001
Never	1323 (46.1)	4816 (60.1)		757 (42.2)	1855 (49.8)		556 (52.5)	2961 (69.0)	
1–14	216 (7.5)	555 (6.9)		163 (9.1)	389 (10.4)		53 (4.9)	166 (3.9)	
15–29	452 (15.7)	889 (11.1)		322 (18.0)	521 (14.0)		130 (12.1)	368 (8.6)	
30–44	449 (15.6)	872 (10.9)		315 (17.6)	520 (14.0)		134 (12.4)	352 (8.2)	
45 or longer	279 (9.7)	537 (6.7)		182 (10.2)	317 (8.5)		97 (9.0)	220 (5.1)	
Missing	152 (5.3)	350 (4.4)		54 (3.0)	125 (3.4)		98 (9.1)	225 (5.2)	
Intensity of ETS exposure			< 0.001			< 0.001			< 0.001
Never	1323 (46.1)	4816 (60.1)		757 (42.2)	1855 (49.8)		556 (52.5)	2961 (69.0)	
Light	560 (19.5)	1212 (15.1)		376 (21.0)	755 (20.3)		184 (17.1)	457 (10.7)	
Medium	493 (17.2)	1030 (12.8)		359 (20.0)	643 (17.3)		134 (12.4)	387 (9.0)	
Heavy	327 (11.4)	580 (7.2)		234 (13.1)	333 (8.9)		93 (8.6)	247 (5.8)	
Missing	168 (5.9)	381 (4.8)		67 (3.7)	141 (3.8)		101 (9.4)	240 (5.6)	
Household ventilation			< 0.001			< 0.001			< 0.001
Good	1106 (38.5)	3418 (42.6)		687 (38.3)	1505 (40.4)		419 (38.9)	1913 (44.6)	
Poor	1711 (59.6)	4539 (56.6)		1073 (59.8)	2197 (59.0)		638 (59.2)	2342 (54.6)	
Missing	54 (1.9)	62 (0.8)		33 (1.8)	25 (0.7)		21 (2.0)	37 (0.9)	
Hot cooking oil			0.009			0.061			0.784
No	1584 (55.2)	4686 (58.4)		942 (52.5)	2,080 (55.8)		642 (59.6)	2606 (60.7)	
Yes	949 (33.1)	2483 (31.0)		605 (33.7)	1152 (30.9)		344 (31.9)	1331 (31.0)	
Missing	338 (11.8)	850 (10.6)		246 (13.7)	495 (13.3)		92 (8.5)	355 (8.3)	
Coal used for cooking			< 0.001			< 0.001			< 0.001
No	1572 (54.8)	5244 (65.4)		1020 (56.9)	2493 (66.9)		552 (51.2)	2751 (64.1)	
Yes	1299 (45.3)	2775 (34.6)		773 (43.1)	1,234 (33.1)		526 (48.8)	1541 (35.9)	
Solid fuel used for heating			0.062			0.734			0.006
No	1027 (35.8)	2754 (34.3)		626 (34.9)	1,309 (35.1)		401 (37.2)	1445 (33.7)	
Yes	1810 (63.0)	5200 (64.9)		1151 (64.2)	2392 (64.2)		659 (61.1)	2808 (65.4)	
Missing	34 (1.2)	65 (0.8)		16 (0.9)	26 (0.7)		18 (1.7)	39 (0.9)	
Number of pollutant sources^b^			< 0.001			< 0.001			< 0.001
0	68 (2.4)	316 (3.9)		29 (1.6)	104 (2.8)		39 (3.6)	212 (4.9)	
1	309 (10.8)	1256 (15.7)		177 (9.9)	537 (14.4)		132 (12.2)	719 (16.8)	
2	710 (24.7)	2381 (29.7)		442 (24.7)	1019 (27.3)		268 (24.9)	1362 (31.7)	
3	746 (26.0)	2018 (25.2)		465 (25.9)	961 (25.8)		281 (26.1)	1057 (24.6)	
4	476 (16.6)	838 (10.5)		317 (17.7)	450 (12.1)		159 (14.8)	388 (9.0)	
5	115 (4.0)	137 (1.7)		73 (4.1)	88 (2.4)		42 (3.9)	49 (1.1)	
Missing	447 (15.6)	1073 (13.4)		290 (16.2)	568 (15.2)		157 (14.6)	505 (11.8)	

a *p* values from *χ*^2^ test.

b Including poor ventilation, hot cooking oil, coal used for cooking, solid fuel used for heating, and ETS exposure.

**Table 2:** Associations between indoor air pollution factors and lung cancer by tobacco smoking status.

	Adjusted ORs (95% CI)		
	All participants^a^ (*n* = 10,890)	Ever smokers^b^ (*n* = 5520)	Never smokers^c^ (*n* = 5370)
Environmental tobacco smoking (ETS)			
Never	Reference	Reference	Reference
Ever	1.54 (1.40, 1.69)	1.32 (1.17, 1.49)	1.84 (1.58, 2.15)
Household ventilation			
Good	Reference	Reference	Reference
Poor	1.18 (1.07, 1.30)	1.15 (1.01, 1.30)	1.24 (1.07, 1.44)
Hot cooking oil			
No	Reference	Reference	Reference
Yes	1.02 (0.93, 1.13)	1.06 (0.93, 1.21)	0.95 (0.82, 1.11)
Coal used for cooking			
No	Reference	Reference	Reference
Yes	1.27 (1.15, 1.41)	1.24 (1.07, 1.43)	1.29 (1.11, 1.51)
Solid fuel used for heating			
No	Reference	Reference	Reference
Yes	1.09 (0.97, 1.21)	1.24 (1.08, 1.44)	0.91 (0.76, 1.08)
Number of indoor air pollution factors^d^			
0	Reference	Reference	Reference
1	0.94 (0.72, 1.21)	0.94 (0.66, 1.36)	0.92 (0.65, 1.32)
2	1.06 (0.83, 1.35)	1.16 (0.82, 1.64)	0.93 (0.66, 1.31)
3	1.25 (0.98, 1.60)	1.28 (0.90, 1.81)	1.18 (0.83, 1.68)
4	1.78 (1.36, 2.32)	1.78 (1.23, 2.58)	1.70 (1.17, 2.49)
5	2.37 (1.68, 3.35)	2.08 (1.30, 3.33)	2.88 (1.69, 4.90)
* p* for trend	< 0.001	< 0.001	< 0.001
WRS of indoor air pollution^e^			
Per IQR increase	1.48 (1.38, 1.60)	1.35 (1.23, 1.48)	1.65 (1.47, 1.85)
Below median	Reference	Reference	Reference
Median or above	1.50 (1.37, 1.65)	1.31 (1.16, 1.49)	1.70 (1.47, 1.97)
Quartile 1	Reference	Reference	Reference
Quartile 2	1.02 (0.87, 1.18)	1.09 (0.88, 1.34)	0.94 (0.75, 1.18)
Quartile 3	1.25 (1.08, 1.45)	1.15 (0.94, 1.41)	1.33 (1.07, 1.65)
Quartile 4	1.74 (1.52, 2.00)	1.52 (1.24, 1.83)	2.04 (1.65, 2.52)
* p* for trend	< 0.001	< 0.001	< 0.001

a Odds ratios adjusted for age, sex, income 10 years ago, education, county of residence, family history of lung cancer, tobacco smoking (ever/never), pack-years of tobacco smoking, alcohol drinking (ever/never), and mutually adjusted for other indoor air pollution factors in this table.

b Odds ratios adjusted for age, sex, income 10 years ago, education, county of residence, family history of lung cancer, pack-years of tobacco smoking, alcohol drinking (ever/never), and mutually adjusted for other indoor air pollution factors in this table.

c Odds ratios adjusted for age, sex, income 10 years ago, education, county of residence, family history of lung cancer, alcohol drinking (ever/never), and mutually adjusted for other indoor air pollution factors in this table.

d Including poor ventilation, hot cooking oil, coal used for cooking, solid fuel used for heating, and ETS exposure.

e Sum of effect estimates for poor ventilation, hot cooking oil, coal used for cooking, solid fuel used for heating, and ETS exposure in the model among all participants. Among all controls the 25^th^ percentile, median, and the 75^th^ percentile are 0.1874, 0.4049, and 0.5823, respectively. The IQR is 0.3949.

## Data Availability

The data that support the findings of this study are available from the corresponding author upon reasonable request.
