# Supplementary material for: The Association Between Indoor Air Pollution and Lung Cancer Risk in a Chinese Population
Source: Indoor Air. Author manuscript; Available in PMC 2026 Feb 27. (PMC11970622; doi:10.1155/ina/9937960)
Supplement: Supplementary material [file NIHMS2068503-supplement-Supplementary_material.docx]

**SUPPLEMENTARY MATERIAL**

**The Association between Indoor Air Pollution and Lung Cancer Risk in a Chinese Population**

Fang Fang, Jin-Yi Zhou, Claire H. Kim, Zi-Yi Jin, Xing Liu, Liming Li, Lina Mu, Ming Wu, Jin-Kou Zhao, Zuo-Feng Zhang

**Table of contents**

Table S1. Associations between exposure to environmental tobacco smoking (ETS) and lung cancer by tobacco smoking status.

Table S2. Associations between indoor air pollution and lung cancer by sex.

Table S3. Joint effect between indoor air pollution and sex on lung cancer by tobacco smoking status.

Table S4. Associations between indoor air pollution factors and lung cancer by tobacco smoking status additionally adjust for proxy interview.

Table S5. Associations between indoor air pollution factors and lung cancer by tobacco smoking status. excluding proxy interviewers.

Table S6. Pearson correlation between predictor variables.

**Table S1. Associations between exposure to environmental tobacco smoking (ETS) and lung cancer by tobacco smoking status.**

|  | All participants (n = 10,890) | Ever smokers (n = 5,520) | Never smokers (n = 5,370) |
| --- | --- | --- | --- |
|  | Adjusted OR*^a^* (95% CI) | Adjusted OR*^b^* (95% CI) | Adjusted OR*^c^* (95% CI) |
| ETS exposure |  |  |  |
| Never | Reference | Reference | Reference |
| Ever | 1.54 (1.40, 1.69) | 1.32 (1.17, 1.49) | 1.84 (1.58, 2.15) |
| Location of ETS exposure |  |  |  |
| Never | Reference | Reference | Reference |
| Home only | 1.57 (1.41, 1.74) | 1.30 (1.13, 1.49) | 1.95 (1.65, 2.30) |
| Work only | 1.18 (0.97, 1.44) | 1.17 (0.94, 1.46) | 0.91 (0.55, 1.53) |
| Both home and work | 1.73 (1.47, 2.04) | 1.49 (1.24, 1.81) | 2.20 (1.58, 3.06) |
| Duration of ETS exposure (years) |  |  |  |
| Never | Reference | Reference | Reference |
| 1-14 | 1.23 (1.04, 1.47) | 1.03 (0.84, 1.27) | 1.83 (1.33, 2.51) |
| 15-29 | 1.58 (1.38, 1.81) | 1.40 (1.18, 1.66) | 1.78 (1.41, 2.26) |
| 30-44 | 1.60 (1.40, 1.83) | 1.40 (1.18, 1.66) | 1.80 (1.44, 2.26) |
| 45 or longer | 1.71 (1.44, 2.02) | 1.41 (1.14, 1.74) | 2.04 (1.55, 2.70) |
| *P for trend* | < 0.001 | < 0.001 | < 0.001 |
| Intensity of ETS exposure |  |  |  |
| Never | Reference | Reference | Reference |
| Light | 1.40 (1.24, 1.59) | 1.14 (0.98, 1.33) | 1.88 (1.54, 2.29) |
| Medium | 1.53 (1.34, 1.74) | 1.34 (1.14, 1.58) | 1.72 (1.37, 2.16) |
| Heavy | 1.90 (1.62, 2.23) | 1.72 (1.41, 2.10) | 2.01 (1.52, 2.65) |
| *P for trend* | < 0.001 | < 0.001 | < 0.001 |

*^a^*Odds ratios are adjusted for age, sex, income 10 years ago, education, county of residence, tobacco smoking (ever/never), tobacco smoking pack-years, alcohol drinking (ever/never) and family history of lung cancer.

*^b^*Odds ratios are adjusted for age, sex, income 10 years ago, education, county of residence, tobacco smoking pack-years, alcohol drinking (ever/never) and family history of lung cancer.

*^c^*Odds ratios are adjusted for age, sex, income 10 years ago, education, county of residence, alcohol drinking (ever/never) and family history of lung cancer.

**Table S2 Associations between indoor air pollution and lung cancer by sex.**

|  | Adjusted ORs (95% CI) | | | | | |
| --- | --- | --- | --- | --- | --- | --- |
|  | All participants^a^ | | Ever smokers^b^ | | Never smokers^c^ | |
|  | Male | Female | Male | Female | Male | Female |
| ETS exposure | | | | | | |
| Never | Reference | Reference | Reference | Reference | Reference | Reference |
| Ever | 1.37 (1.23, 1.54) | 1.76 (1.48, 2.09) | 1.30 (1.15, 1.48) | 1.44 (0.99, 2.09) | 1.64 (1.26, 2.14) | 1.88 (1.55, 2.29) |
| Household ventilation | | | | | | |
| Good | Reference | Reference | Reference | Reference | Reference | Reference |
| Poor | 1.18 (1.05, 1.32) | 1.17 (0.98, 1.39) | 1.18 (1.03, 1.35) | 0.84 (0.57, 1.22) | 1.17 (0.93, 1.47) | 1.25 (1.03, 1.51) |
| Hot cooking oil | | | | | | |
| No | Reference | Reference | Reference | Reference | Reference | Reference |
| Yes | 1.04 (0.92, 1.17) | 1.00 (0.84, 1.18) | 1.07 (0.93, 1.24) | 0.98 (0.66, 1.45) | 0.90 (0.70, 1.15) | 0.98 (0.81, 1.20) |
| Coal used for cooking | | | | | | |
| No | Reference | Reference | Reference | Reference | Reference | Reference |
| Yes | 1.20 (1.06, 1.37) | 1.44 (1.19, 1.74) | 1.23 (1.06, 1.44) | 1.34 (0.79, 2.25) | 1.08 (0.85, 1.37) | 1.47 (1.20, 1.80) |
| Solid fuel used for heating | | | | | | |
| No | Reference | Reference | Reference | Reference | Reference | Reference |
| Yes | 1.15 (1.01, 1.32) | 0.97 (0.79, 1.18) | 1.21 (1.04, 1.40) | 1.73 (1.07, 2.79) | 0.99 (0.74, 1.33) | 0.86 (0.69, 1.07) |
| Number of indoor air factors^d^ | | | | | | |
| 3 or less | Reference | Reference | Reference | Reference | Reference | Reference |
| 4 or more | 1.33 (1.19, 1.50) | 1.53 (1.29, 1.82) | 1.35 (1.18, 1.55) | 1.23 (0.84, 1.80) | 1.24 (0.98, 1.58) | 1.62 (1.33, 1.97) |
| WRS of indoor air pollutantion^e^ | | | | | | |
| Below median | Reference | Reference | Reference | Reference | Reference | Reference |
| Median or above | 1.35 (1.20, 1.52) | 1.77 (1.49, 2.11) | 1.28 (1.12, 1.46) | 1.65 (1.11, 2.44) | 1.50 (1.20, 1.89) | 1.83 (1.50. 2.22) |

^a^Odds ratios adjusted for age, income 10 years ago, education, county of residence, family history of lung cancer, tobacco smoking (ever/never), pack-years of tobacco smoking, and alcohol drinking (ever/never).

^b^Odds ratios adjusted for age, income 10 years ago, education, county of residence, family history of lung cancer, pack-years of tobacco smoking, and alcohol drinking (ever/never).

^c^Odds ratios adjusted for age, income 10 years ago, education, county of residence, family history of lung cancer, and alcohol drinking (ever/never).

^d^Including poor ventilation, hot cooking oil, coal used for cooking, solid fuel used for heating, and ETS exposure.

^e^Sum of effect estimates for poor ventilation, hot cooking oil, coal used for cooking, solid fuel used for heating, and ETS exposure in the model among all participants. Among all controls the median is 0.4049.

**Table S3. Joint effect between indoor air pollution and sex on lung cancer by tobacco smoking status.**

|  | Adjusted ORs (95% CI) | | | | | |
| --- | --- | --- | --- | --- | --- | --- |
|  | All participants^a^ | | Ever smokers^b^ | | Never smokers^c^ | |
|  | Male | Female | Male | Female | Male | Female |
| ETS exposure | | | | | | |
| Never | Reference | 1.62 (1.39, 1.89) | Reference | 1.30 (0.94, 1.80) | Reference | 1.81 (1.50, 2.19) |
| Ever | 1.42 (1.27, 1.59) | 2.97 (2.55, 3.46) | 1.31 (1.15, 1.48) | 1.91 (1.48, 2.46) | 1.68 (1.30, 2.18) | 3.50 (2.85, 4.31) |
| RERI | 0.93 (0.50, 1.35) | | 0.30 (-0.29, 0.89) | | 1.01 (0.34, 1.67) | |
| *p for RERI* | < 0.001 | | 0.320 | | 0.003 | |
| ROR | 1.29 (1.06, 1.57) | | 1.12 (0.76, 1.66) | | 1.15 (0.84, 1.57) | |
| *p for ROR* | 0.013 | | 0.565 | | 0.389 | |
| Number of indoor air pollution factors^d^ | | | | | | |
| 3 or less | Reference | 1.61 (1.38, 1.88) | Reference | 1.36 (1.03, 1.81) | Reference | 1.66 (1.35, 2.03) |
| 4 or more | 1.26 (1.12, 1.41) | 2.91 (2.49, 3.40) | 1.33 (1.16, 1.52) | 1.99 (1.51, 2.62) | 1.10 (0.88, 1.39) | 2.96 (2.40, 3.66) |
| RERI | 1.04 (0.63, 1.45) | | 0.29 (-0.33, 0.91) | | 1.20 (0.70, 1.70) | |
| *p for RERI* | < 0.001 | | 0.354 | | < 0.001 | |
| ROR | 1.43 (1.18, 1.75) | | 1.10 (0.75, 1.60) | | 1.62 (1.21, 2.16) | |
| *p for ROR* | < 0.001 | | 0.634 | | 0.001 | |
| WRS of indoor air pollution^e^ | | | | | | |
| Below median | Reference | 1.50 (1.27, 1.79) | Reference | 1.19 (0.83, 1.69) | Reference | 1.71 (1.37, 2.14) |
| Median or above | 1.34 (1.20, 1.50) | 2.90 (2.50, 3.36) | 1.28 (1.12, 1.46) | 1.95 (1.52, 2.50) | 1.45 (1.15, 1.81) | 3.28 (2.66, 4.04) |
| RERI | 1.05 (0.66, 1.44) | | 0.48 (-0.10, 1.07) | | 1.12 (0.59, 1.66) | |
| *p for RERI* | < 0.001 | | 0.103 | | < 0.001 | |
| ROR | 1.44 (1.17, 1.76) | | 1.28 (0.85, 1.94) | | 1.32 (0.99, 1.77) | |
| *p for ROR* | < 0.001 | | 0.234 | | 0.059 | |

^a^Odds ratios adjusted for age, income 10 years ago, education, county of residence, family history of lung cancer, tobacco smoking (ever/never), pack-years of tobacco smoking, and alcohol drinking (ever/never).

^b^Odds ratios adjusted for age, income 10 years ago, education, county of residence, family history of lung cancer, pack-years of tobacco smoking, and alcohol drinking (ever/never).

^c^Odds ratios adjusted for age, income 10 years ago, education, county of residence, family history of lung cancer, and alcohol drinking (ever/never).

^d^Including poor ventilation, hot cooking oil, coal used for cooking, solid fuel used for heating, and ETS exposure.

^e^Sum of effect estimates for poor ventilation, hot cooking oil, coal used for cooking, solid fuel used for heating, and ETS exposure in the model among all participants. Among all controls the median is 0.4049.

**Table S4. Associations between indoor air pollution factors and lung cancer by tobacco smoking status additionally adjust for proxy interview.**

|  | Adjusted ORs (95% CI) | | |
| --- | --- | --- | --- |
|  | All participants*^a^*  (n = 10,890) | Ever smokers*^b^*  (n = 5,520) | Never smokers*^c^*  (n = 5,370) |
| Environmental tobacco smoking (ETS) |  |  |  |
| Never | Reference | Reference | Reference |
| Ever | 1.40 (1.27, 1.55) | 1.23 (1.08, 1.40) | 1.64 (1.39, 1.93) |
| Household ventilation |  |  |  |
| Good | Reference | Reference | Reference |
| Poor | 1.18 (1.07, 1.31) | 1.17 (1.02, 1.33) | 1.24 (1.05, 1.45) |
| Hot cooking oil |  |  |  |
| No | Reference | Reference | Reference |
| Yes | 1.02 (0.92 1.14) | 1.06 (0.92, 1.21) | 0.96 (0.81, 1.13) |
| Coal used for cooking |  |  |  |
| No | Reference | Reference | Reference |
| Yes | 1.23 (1.10, 1.38) | 1.23 (1.05, 1.44) | 1.22 (1.03, 1.45) |
| Solid fuel used for heating |  |  |  |
| No | Reference | Reference | Reference |
| Yes | 1.06 (0.94, 1.20) | 1.19 (1.02, 1.39) | 0.89 (0.74, 1.08) |
| Number of indoor air pollution factors*^d^* |  |  |  |
| 0 | Reference | Reference | Reference |
| 1 | 0.88 (0.67, 1.15) | 0.88 (0.60, 1.29) | 0.89 (0.60, 1.30) |
| 2 | 1.02 (0.79, 1.32) | 1.14 (0.79, 1.63) | 0.88 (0.60, 1.27) |
| 3 | 1.17(0.91, 1.52) | 1.22 (0.84, 1.76) | 1.10 (0.75, 1.59) |
| 4 | 1.59 (1.20, 2.10) | 1.65 (1.12, 2.43) | 1.45 (0.96, 2.20) |
| 5 | 1.96 (1.35, 2.85) | 1.67 (1.01, 2.76) | 2.58 (1.45, 4.60) |
| *p for trend* | < 0.001 | < 0.001 | < 0.001 |
| WRS of indoor air pollution*^e^* |  |  |  |
| Per IQR increase | 1.39 (1.29, 1.51) | 1.329 (1.17, 1.42) | 1.52 (1.34, 1.72) |
| Below median | Reference | Reference | Reference |
| Median or above | 1.37 (1.24, 1.51) | 1.23 (1.08, 1.41) | 1.51 (1.29, 1.77) |
| Quartile 1 | Reference | Reference | Reference |
| Quartile 2 | 1.09 (0.93, 1.27) | 1.19 (0.96, 1.48) | 0.97 (0.76, 1.23) |
| Quartile 3 | 1.18 (1.01, 1.38) | 1.15 (0.93, 1.41) | 1.18 (0.93, 1.49) |
| Quartile 4 | 1.64 (1.42, 1.90) | 1.49 (1.24, 1.80) | 1.85 (1.47, 2.32) |
| *p for trend* | <0.001 | < 0.001 | < 0.001 |

*^a^*Odds ratios adjusted for age, sex, income 10 years ago, education, county of residence, family history of lung cancer, tobacco smoking (ever/never), pack-years of tobacco smoking, alcohol drinking (ever/never), proxy interview (yes/no) and mutually adjusted for other indoor air pollution factors in this table.

*^b^*Odds ratios adjusted for age, sex, income 10 years ago, education, county of residence, family history of lung cancer, pack-years of tobacco smoking, alcohol drinking (ever/never), proxy interview (yes/no) and mutually adjusted for other indoor air pollution factors in this table.

*^c^*Odds ratios adjusted for age, sex, income 10 years ago, education, county of residence, family history of lung cancer, alcohol drinking (ever/never), proxy interview (yes/no) and mutually adjusted for other indoor air pollution factors in this table.

*^d^*Including poor ventilation, hot cooking oil, coal used for cooking, solid fuel used for heating, and ETS exposure.

*^e^*Sum of effect estimates for poor ventilation, hot cooking oil, coal used for cooking, solid fuel used for heating, and ETS exposure in the model among all participants. Among all controls the 25^th^ percentile, median, and the 75^th^ percentile are 0.1874, 0.4049, and 0.5823, respectively. The IQR is 0.3949.

**Table S5. Associations between indoor air pollution factors and lung cancer by tobacco smoking status excluding proxy interviewers.**

|  | Adjusted ORs (95% CI) | | |
| --- | --- | --- | --- |
|  | All participants*^a^*  (n = 9,124) | Ever smokers*^b^*  (n = 4,688) | Never smokers*^c^*  (n = 5,370) |
| Environmental tobacco smoking (ETS) |  |  |  |
| Never | Reference | Reference | Reference |
| Ever | 1.37 (1.22, 1.53) | 1.22 (1.06, 1.42) | 1.53 (1.25, 1.87) |
| Household ventilation |  |  |  |
| Good | Reference | Reference | Reference |
| Poor | 1.19 (1.06, 1.34) | 1.07 (0.92, 1.24) | 1.18 (0.97, 1.42) |
| Hot cooking oil |  |  |  |
| No | Reference | Reference | Reference |
| Yes | 1.04 (0.92 1.17) | 1.15 (0.97, 1.37) | 0.96 (0.79, 1.17) |
| Coal used for cooking |  |  |  |
| No | Reference | Reference | Reference |
| Yes | 1.20 (1.05, 1.36) | 1.17 (0.99, 1.39) | 1.26 (1.02, 1.55) |
| Solid fuel used for heating |  |  |  |
| No | Reference | Reference | Reference |
| Yes | 1.07 (0.94, 1.23) | 1.24 (1.08, 1.42) | 0.92 (0.74, 1.16) |
| Number of indoor air pollution factors*^d^* |  |  |  |
| 0 | Reference | Reference | Reference |
| 1 | 0.82 (0.62, 1.10) | 0.89 (0.59, 1.34) | 0.78 (0.51, 1.16) |
| 2 | 0.98 (0.75, 1.29) | 1.18 (0.81, 1.72) | 0.78 (0.53, 1.15) |
| 3 | 1.13(0.86, 1.49) | 1.28 (0.87, 1.89) | 0.96 (0.64, 1.44) |
| 4 | 1.50 (1.11, 2.02) | 1.66 (1.10, 2.50) | 1.30 (0.83, 2.06) |
| 5 | 1.95 (1.27, 2.99) | 1.69 (0.96, 2.98) | 2.63 (1.36, 5.07) |
| *p for trend* | < 0.001 | < 0.001 | < 0.001 |
| WRS of indoor air pollution*^e^* |  |  |  |
| Per IQR increase | 1.36 (1.25, 1.49) | 1.28 (1.15, 1.42) | 1.46 (1.26, 1.70) |
| Below median | Reference | Reference | Reference |
| Median or above | 1.31 (1.17, 1.47) | 1.20 (1.04, 1.38) | 1.42 (1.17, 1.72) |
| Quartile 1 | Reference | Reference | Reference |
| Quartile 2 | 1.07 (0.90, 1.28) | 1.22 (0.97, 1.53) | 0.90 (0.69, 1.19) |
| Quartile 3 | 1.09 (0.92, 1.30) | 1.10 (0.88, 1.38) | 1.05 (0.80, 1.39) |
| Quartile 4 | 1.58 (1.34, 1.85) | 1.50 (1.22, 1.84) | 1.69 (1.30, 2.20) |
| *p for trend* | <0.001 | < 0.001 | < 0.001 |

*^a^*Odds ratios adjusted for age, sex, income 10 years ago, education, county of residence, family history of lung cancer, tobacco smoking (ever/never), pack-years of tobacco smoking, alcohol drinking (ever/never), proxy interview (yes/no) and mutually adjusted for other indoor air pollution factors in this table.

*^b^*Odds ratios adjusted for age, sex, income 10 years ago, education, county of residence, family history of lung cancer, pack-years of tobacco smoking, alcohol drinking (ever/never), proxy interview (yes/no) and mutually adjusted for other indoor air pollution factors in this table.

*^c^*Odds ratios adjusted for age, sex, income 10 years ago, education, county of residence, family history of lung cancer, alcohol drinking (ever/never), proxy interview (yes/no) and mutually adjusted for other indoor air pollution factors in this table.

*^d^*Including poor ventilation, hot cooking oil, coal used for cooking, solid fuel used for heating, and ETS exposure.

*^e^*Sum of effect estimates for poor ventilation, hot cooking oil, coal used for cooking, solid fuel used for heating, and ETS exposure in the model among all participants. Among all controls the 25^th^ percentile, median, and the 75^th^ percentile are 0.1874, 0.4049, and 0.5823, respectively. The IQR is 0.3949.

**Table S6. Pearson correlation between predictor variables.**

|  | Environmental tobacco smoking (ETS) | Household ventilation | Hot cooking oil | Coal used for cooking | Solid fuel used for heating | Age | Sex | Income 10 years ago | Education | County of residence | Family history of lung cancer | Tobacco smoking | Alcohol drinking |
| --- | --- | --- | --- | --- | --- | --- | --- | --- | --- | --- | --- | --- | --- |
| ETS | 1.000 | 0.005 | 0.050 | 0.047 | -0.051 | 0.013 | 0.034 | 0.010 | -0.022 | -0.158 | 0.038 | 0.203 | 0.138 |
| Household ventilation |  | 1.000 | < 0.001 | 0.017 | 0.084 | 0.167 | -0.001 | -0.121 | -0.185 | -0.042 | -0.028 | 0.038 | -0.005 |
| Hot cooking oil |  |  | 1.000 | 0.021 | 0.005 | -0.017 | 0.012 | 0.009 | -0.008 | 0.015 | 0.014 | 0.018 | 0.018 |
| Coal used for cooking |  |  |  | 1.000 | 0.075 | -0.025 | -0.012 | -0.016 | 0.005 | 0.345 | -0.041 | -0.022 | 0.006 |
| Solid fuel used for heating |  |  |  |  | 1.000 | 0.003 | -0.021 | -0.078 | -0.004 | -0.088 | 0.007 | -0.006 | 0.017 |
| Age |  |  |  |  |  | 1.000 | <0.001 | -0.132 | -0.452 | 0.009 | -0.038 | 0.066 | -0.025 |
| Sex |  |  |  |  |  |  | 1.000 | -0.011 | -0.263 | 0.044 | 0.009 | -0.406 | -0.431 |
| Income 10 years ago |  |  |  |  |  |  |  | 1.000 | 0.169 | -0.101 | 0.002 | -0.022 | 0.012 |
| Education |  |  |  |  |  |  |  |  | 1.000 | 0.015 | 0.036 | 0.028 | 0.114 |
| County of residence |  |  |  |  |  |  |  |  |  | 1.000 | -0.057 | -0.253 | -0.214 |
| Family history of lung cancer |  |  |  |  |  |  |  |  |  |  | 1.000 | 0.034 | 0.011 |
| Tobacco smoking |  |  |  |  |  |  |  |  |  |  |  | 1.000 | 0.450 |
| Alcohol drinking |  |  |  |  |  |  |  |  |  |  |  |  | 1.000 |
